# Multidimensional Social Interaction and School Refusal Tendency Among Elementary and Junior High School Students: A Three-Year Longitudinal Study with an Exploratory Indirect-Association Analysis

**DOI:** 10.3390/children13081079

**Published:** 2026-08-14

**Authors:** Ruifeng Zhao, Haotian Gao, Yixin Sun, Mengxuan Wang, Shuanghong Li, Jinrui Zhang, Ruoyu Zhou, Yanlin Wang, Ying Huang, Yuko Sawada, Akihiro Kakuda, Tokie Anme

**Affiliations:** 1 School of Future Education, Qingdao Hengxing University of Science and Technology, Qingdao 266100, China; zhao43218611@gmail.com (R.Z.); zhouruoyu86@gmail.com (R.Z.); 2 Doctoral Program in Medical Sciences, Graduate School of Comprehensive Human Sciences, University of Tsukuba, Tsukuba 305-8577, Japan; s2330446@u.tsukuba.ac.jp (H.G.); sunyixin10@outlook.com (Y.S.); s2230446@u.tsukuba.ac.jp (M.W.); s2330440@u.tsukuba.ac.jp (S.L.); jinrui970308@gmail.com (J.Z.); violin1113600@gmail.com (Y.W.); huangyvonne11@gmail.com (Y.H.); 3 Department of Physical Therapy, Morinomiya University of Medical Sciences, Osaka 559-8611, Japan; ysawa1110@yahoo.co.jp (Y.S.); kakuda@morinomiya-u.ac.jp (A.K.); 4 Faculty of Medicine, University of Tsukuba, 1-1-1 Tennodai, Tsukuba 305-8575, Japan

**Keywords:** school refusal tendency, multidimensional social interaction, Index of Social Interaction, Strengths and Difficulties Questionnaire, psychological and behavioral difficulties, internalizing problems, peer problems, adolescents, longitudinal study, Japan

## Abstract

**Highlights:**

**What are the main findings?**

- Higher baseline social interaction was associated with lower psychological and behavioral difficulties and lower odds of school refusal tendency three years later.
- Psychological and behavioral difficulties, particularly internalizing-related difficulties, were associated with a significant exploratory indirect effect linking baseline social interaction and later school refusal tendency.

**What are the implications of the main findings?**

- Assessment of social engagement and SDQ-based difficulties may help identify domains that warrant further evaluation and support.
- Community- and school-based initiatives may support social engagement, emotional adjustment, peer relationships, and trusted interpersonal connections.

**Abstract:**

Background/Objectives: School refusal tendency is an important school adaptation issue, but its upstream psychosocial correlates remain insufficiently understood. We examined whether baseline multidimensional social interaction was prospectively associated with later school refusal tendency and whether an exploratory statistical indirect association was observed through psychological and behavioral difficulties. Methods: This three-year longitudinal observational study included 314 students in grades 5–9 aged ≥12 years at baseline in 2020 and followed through 2023. Social interaction was assessed using the Index of Social Interaction (ISI), psychological and behavioral difficulties using the parent-reported Strengths and Difficulties Questionnaire (SDQ), and school refusal tendency using a parent-reported binary item. Adjusted regression and Monte Carlo product-of-coefficients analyses were conducted. Results: School refusal tendency was observed in 84 participants (26.8%). Higher baseline ISI scores were associated with lower follow-up SDQ total difficulties (β = −0.830, 95% CI: −1.033 to −0.627) and lower odds of school refusal tendency (OR = 0.832, 95% CI: 0.741–0.934). The indirect effect through follow-up SDQ total difficulties was significant (−0.120, 95% CI: −0.193 to −0.055). After adjustment for baseline SDQ total difficulties, the indirect effect remained significant (−0.067, 95% CI: −0.124 to −0.020); Firth and parsimonious models yielded similar estimates. Baseline-domain-adjusted analyses retained indirect effects through emotional symptoms, peer problems, and internalizing problems. Conclusions: Higher baseline multidimensional social interaction was associated with lower subsequent psychological and behavioral difficulties and school refusal tendency. Baseline-SDQ-adjusted analyses supported an exploratory indirect association, particularly through internalizing-related difficulties; however, concurrent assessment of follow-up SDQ and school refusal precludes causal interpretation.

## 1. Introduction

School refusal and school attendance problems have become important issues in child and adolescent health. School refusal is generally understood as difficulty attending school or reluctance to attend school that is not primarily explained by antisocial behavior or parental neglect [1]. Although the severity of school refusal varies, reluctance to attend school may represent an early sign of school maladjustment before frequent or prolonged absence becomes evident [2,3]. School refusal is not only an educational concern but also a public health and developmental issue, as it is often associated with psychological distress, peer relationship difficulties, family stress, and impaired social functioning [4,5]. In Japan, school refusal has drawn increasing attention in recent years, particularly among elementary and junior high school students, highlighting the need to identify early psychosocial factors associated with school maladjustment [6].

Psychological and behavioral difficulties are among the most consistently reported correlates of school refusal and absenteeism. Children and adolescents who experience emotional symptoms, anxiety, depressive tendencies, peer problems, behavioral difficulties, or attention-related problems may find it more difficult to participate in school life and maintain stable attendance [7,8]. Among these difficulties, internalizing problems, such as emotional symptoms and peer relationship problems, may be particularly relevant to school refusal tendency, because reluctance to attend school often reflects emotional distress or interpersonal difficulties rather than overt behavioral problems alone [1,9]. Therefore, examining psychological and behavioral difficulties may help clarify the pathway through which broader psychosocial factors contribute to later school refusal tendency [10].

The Strengths and Difficulties Questionnaire (SDQ) is widely used to assess psychological and behavioral difficulties in children and adolescents [11]. It includes five subdomains: emotional symptoms, conduct problems, hyperactivity/inattention, peer problems, and prosocial behavior. The SDQ total difficulties score, calculated from the four difficulty subdomains, provides a brief indicator of overall psychological and behavioral difficulties. Broader internalizing and externalizing problem scores can also be calculated, which may be useful in general population samples [12,13]. The Japanese version of the SDQ has been validated in community and school-aged child samples, supporting its use as a screening tool for psychological and behavioral difficulties among Japanese children [14,15]. Because the SDQ captures multiple dimensions of child functioning, it is suitable for evaluating whether a statistical indirect association is observed between earlier social conditions and later school-related outcomes through psychological and behavioral difficulties.

Social interaction is another important factor in child and adolescent development. In a narrow sense, social interaction generally refers to communication with others, interpersonal relationships, and participation in social activities [16,17]. These core aspects may contribute to psychological adjustment by supporting emotional regulation, peer connection, social learning, and a sense of belonging. In community health research, however, social interaction is often conceptualized more broadly to include related psychosocial dimensions that reflect how individuals engage with their surrounding social environment [18]. In this broader framework, social curiosity and daily independence may be viewed as related dimensions of active social engagement rather than as core elements of social interaction itself. Perceived safety and trust, meanwhile, represent the quality and reliability of interpersonal and community support, which may shape children’s willingness to interact with others and participate in school or community life [19,20]. Conversely, limited social interaction and weaker social engagement may increase vulnerability to emotional symptoms, peer problems, and reduced prosocial behavior, which may in turn affect school adaptation [21,22]. From a community health perspective, social interaction can therefore be viewed as an upstream psychosocial resource that may influence later mental health and school functioning [23].

The Index of Social Interaction (ISI) is a community-based measure developed to assess multiple aspects of social interaction in community-dwelling populations across the life course. It consists of 18 items covering interaction, participation, social curiosity, independence, and safety/trust. Previous community-based studies in Japan have supported the reliability and validity of the ISI, including acceptable internal consistency and predictive validity for later health-related outcomes [24,25]. Although the ISI has often been applied in adult and older-adult community health research, its multidimensional structure is conceptually applicable to broader community populations, including adolescents aged 12 years and older [26]. In the present study, the ISI was used as a baseline indicator of social interaction among elementary and junior high school students.

Despite growing attention to school refusal and child mental health, several research gaps remain. First, many previous studies have focused on psychological symptoms after school refusal has already emerged, whereas fewer studies have examined upstream social factors that may precede later school refusal tendency. Second, although social relationships and peer difficulties are known to be important for school adjustment, limited evidence has examined multidimensional social interaction and engagement as a broader community-based factor. Third, few longitudinal studies have evaluated whether an exploratory statistical indirect association links earlier social interaction with later school refusal tendency through psychological and behavioral difficulties. Whereas much of the earlier evidence has been cross-sectional, the present study temporally separated baseline social interaction from psychological and school-related outcomes assessed three years later and additionally evaluated whether the findings persisted after adjustment for baseline SDQ levels. Addressing these gaps may help identify psychosocial domains that warrant further investigation in community- and school-based research [7,9].

Therefore, this study aimed to examine the prospective association between baseline multidimensional social interaction and later school refusal tendency among elementary and junior high school students in grades 5 through 9 who were aged 12 years or older at baseline in a village in Aichi Prefecture, central Japan, and to determine whether an exploratory statistical indirect association was observed through psychological and behavioral difficulties. Using data from the Community Empowerment and Care for Wellbeing and Healthy Longevity (CEC) study collected in 2020 and 2023, we tested three hypotheses. First, higher baseline social interaction in 2020 would be associated with lower psychological and behavioral difficulties in 2023. Second, higher psychological and behavioral difficulties in 2023 would be associated with higher odds of school refusal tendency in 2023. Third, an indirect association between baseline social interaction and later school refusal tendency would be observed through psychological and behavioral difficulties. Frequent school absence was examined as a supplementary school-related outcome because of its low event count.

## 2. Materials and Methods

### 2.1. Study Design and Setting

This was a three-year longitudinal observational analysis conducted from 2020 to 2023 as part of the Community Empowerment and Care for Wellbeing and Healthy Longevity (CEC) study. The CEC study is an ongoing community-based cohort project designed to examine health, lifestyle, psychosocial functioning, and wellbeing across the lifespan. The present study was conducted in a village in Aichi Prefecture, central Japan. Data collection for each survey wave began in April, coinciding with the start of the new academic year in Japan; the baseline and follow-up surveys began in April 2020 and April 2023, respectively.

The present analysis focused on elementary and junior high school students in grades 5 through 9 who were aged 12 years or older at baseline, participated in the 2020 baseline survey, and were followed up in the 2023 survey. Baseline social interaction, SDQ scores, and covariates were assessed in 2020, whereas follow-up SDQ scores and school-related outcomes were assessed in 2023. No previously published article has used the same child cohort included in the present study; there is no overlap with previous publications in survey years, participants, or analytical samples. The research question, exposure, outcomes, and analytical approach of the present study are also distinct from those of prior CEC publications.

### 2.2. Participants

Participants were selected from students who took part in the 2020 baseline survey of the CEC study. Students were eligible for the present analysis if they were enrolled in grades 5 through 9, were aged 12 years or older at baseline, and had linkable follow-up data in 2023. The age criterion was applied because the Index of Social Interaction (ISI), the primary exposure measure, is intended for individuals aged 12 years or older.

**Figure 1:**
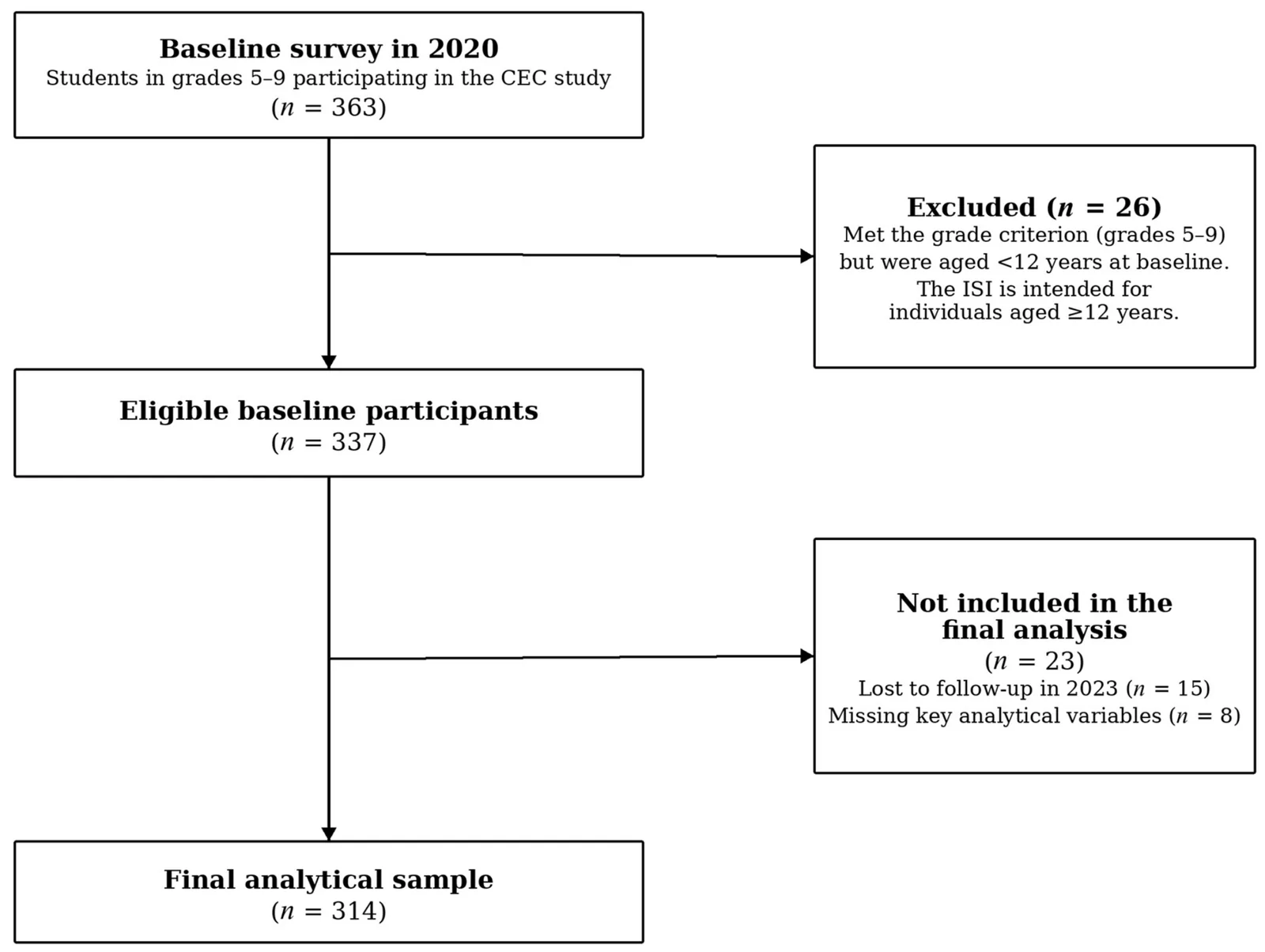
Flow diagram of participant selection. Note. CEC, Community Empowerment and Care for Wellbeing and Healthy Longevity; ISI, Index of Social Interaction. The 26 excluded students met the grade criterion (grades 5–9) but were younger than 12 years at baseline; the ISI is intended for individuals aged 12 years or older.

A total of 363 students in grades 5–9 participated in the 2020 baseline survey. Among them, 26 met the grade criterion but were younger than 12 years at baseline and were therefore excluded from the ISI-based analysis. Of the remaining 337 eligible participants, 15 were lost to follow-up in 2023 and 8 had missing data on key analytical variables, resulting in a final analytical sample of 314 participants (Figure 1).

### 2.3. Ethical Considerations

The study involving human participants was approved by the Medical Ethics Committee of the University of Tsukuba, Japan (approval number: 1331-7; approval date: 12 December 2018). The study was conducted in accordance with the Declaration of Helsinki and relevant institutional and ethical requirements. Approval and cooperation for the survey were obtained from the relevant local government authorities and participating schools. Informed consent was obtained from parents or legal guardians, and assent was obtained from all participating students prior to participation.

### 2.4. Measures

#### 2.4.1. Social Interaction

Social interaction and engagement were assessed at baseline in 2020 using the Index of Social Interaction (ISI) [26]. The ISI is a community-based measure consisting of 18 items across five subdomains: interaction, participation, social curiosity, independence, and safety/trust. Responses were scored according to the official ISI scoring guidelines. For each item, the first three response categories were classified as positive responses and scored 1, whereas the fourth response category was classified as a negative response and scored 0. The 18 item scores were summed to obtain a total score ranging from 0 to 18, with higher scores indicating a higher level of multidimensional social interaction and engagement.

The five ISI subdomain scores were also calculated. The interaction domain reflects communication and interaction with family members and non-family members. The participation domain reflects involvement in social groups, neighborhood activities, and social roles. The social curiosity domain reflects engagement in hobbies, reading, use of new technologies, and interest in society. The independence domain reflects motivation for active living, regular lifestyle, health motivation, and proactive life engagement. The safety/trust domain reflects perceived availability of consultation and emergency support.

The ISI has been used in previous community-based studies in Japan. Its factor structure and predictive validity have been supported in prior community research [24,25,27]. In the present adolescent sample, the 18-item ISI demonstrated acceptable internal consistency (Cronbach’s α = 0.757). The ISI total score in 2020 was used as the primary exposure variable, and the five subdomains were used for descriptive analyses.

#### 2.4.2. Psychological and Behavioral Difficulties

Psychological and behavioral difficulties were assessed at baseline in 2020 and follow-up in 2023 using the parent-reported Japanese version of the Strengths and Difficulties Questionnaire (SDQ). The SDQ is a widely used brief behavioral screening questionnaire for children and adolescents [28,29]. It consists of 25 items covering five subscales: emotional symptoms, conduct problems, hyperactivity/inattention, peer problems, and prosocial behavior. Each subscale consists of five items.

Each SDQ item was scored from 0 to 2 according to the standard scoring method. Positively worded difficulty items were reverse-coded when required. The SDQ total difficulties score was calculated as the sum of four difficulty subscales: emotional symptoms, conduct problems, hyperactivity/inattention, and peer problems. The prosocial behavior subscale was not included in the total difficulties score. Higher total difficulties scores indicate greater psychological and behavioral difficulties, whereas higher prosocial behavior scores indicate more prosocial behavior. In addition to the five original SDQ subscales, internalizing and externalizing problem scores were calculated. Internalizing problems were calculated as the sum of emotional symptoms and peer problems, and externalizing problems were calculated as the sum of conduct problems and hyperactivity/inattention. These broader subscale groupings are often recommended for use in general population samples [30].

The SDQ has demonstrated acceptable reliability and validity in previous international studies and has been widely used in clinical, school, and community-based research [31]. The Japanese version of the SDQ has also been examined in community and school-aged child samples, with previous studies supporting its reliability, validity, and normative interpretation in Japanese children [14,32]. In the primary analysis, the SDQ total difficulties score in 2023 was treated as the candidate intermediate variable. Corresponding baseline SDQ total and domain scores from 2020 were incorporated into sensitivity analyses. Follow-up SDQ subdomain scores, internalizing problems, externalizing problems, and prosocial behavior were evaluated separately in supplementary indirect-association analyses.

#### 2.4.3. School Refusal Tendency and Frequent School Absence

School-related outcomes were assessed in 2023 based on reports from parents or guardians referring to the previous year. School refusal tendency was assessed using the item: “During the past year, were there times when your child did not want to go to school?” Response options were (1) always, (2) often, (3) sometimes, (4) rarely, and (5) never. Responses of always, often, or sometimes (categories 1–3) were classified as the presence of school refusal tendency, whereas rarely or never (categories 4–5) were classified as its absence.

Frequent school absence was assessed using the item: “During the past year, how often did your child miss school?” Response options were (1) almost every day, (2) approximately 1–2 days per week, (3) approximately 1–2 days per month, (4) approximately 1–2 days per six months, and (5) approximately 1–2 days per year or less. Responses of almost every day or approximately 1–2 days per week (categories 1–2) were classified as frequent school absence, whereas categories 3–5 were classified as no frequent school absence. Because the event count was small, frequent school absence was treated as a supplementary exploratory outcome.

Both outcomes were single-item, parent- or guardian-reported questionnaire indicators and were not based on clinical diagnoses or official school attendance records.

#### 2.4.4. Covariates

Baseline covariates were assessed in 2020. These included sex, age, body mass index (BMI), only-child status, exercise habit, good sleep, hospitalization history, parental scolding, and movement disability. BMI was calculated as weight in kilograms divided by height in meters squared (kg/m^2^), using the student’s height and weight at the time of the survey as reported by the parent or guardian; height and weight were not directly measured by the research team. Number of siblings was dichotomized into only-child status. Only-child status, exercise habit, good sleep, hospitalization history, parental scolding, and movement disability were treated as binary variables.

**Figure 2:**
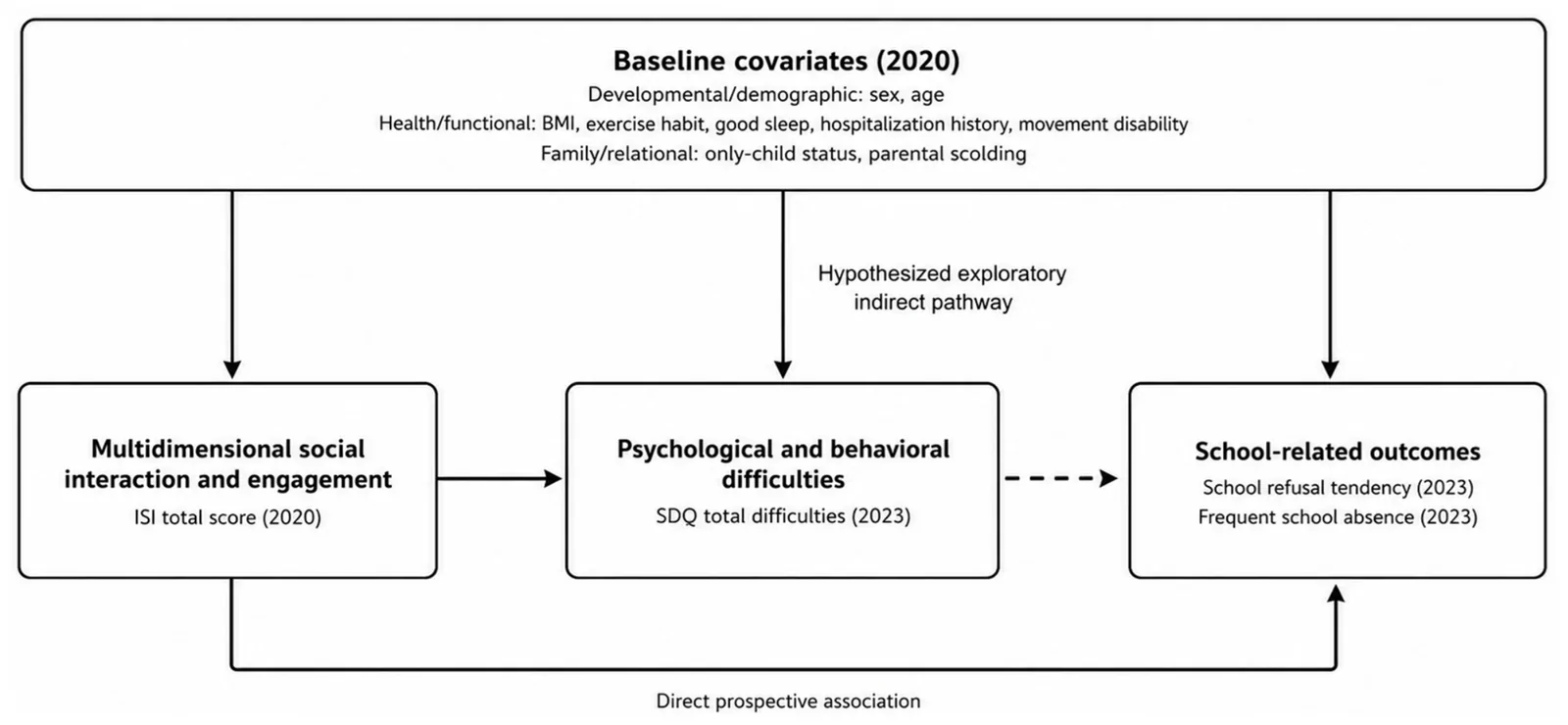
A priori DAG-informed conceptual framework for covariate selection and the exploratory indirect-association analysis. Note. Solid arrows represent hypothesized baseline or prospective relationships. The dashed arrow represents the exploratory association between SDQ and school-related outcomes, which were measured concurrently in 2023. The framework guided covariate adjustment and should not be interpreted as evidence of causal mediation. BMI, body mass index; ISI, Index of Social Interaction; SDQ, Strengths and Difficulties Questionnaire.

Covariates were selected a priori using a directed acyclic graph (DAG)-informed conceptual framework based on temporal ordering and previous literature (Figure 2). Variables measured at baseline that could plausibly influence multidimensional social interaction, subsequent psychological and behavioral difficulties, and school-related outcomes were treated as potential confounders. Sex and age represented developmental and demographic differences. BMI was included because weight status has been associated with child and adolescent mental health [33]. Only-child status and parental scolding represented family and relational context; only-child status has been associated with prosocial behavior and parenting context [34]. Exercise habit, good sleep, hospitalization history, and movement disability represented baseline health and functional factors; movement behaviors may be related to cognitive and social functioning [35,36]. Parental scolding was included as an indicator of adverse parent–child interaction because parental psychological control and related family factors have been associated with emotion regulation, behavioral difficulties, and school refusal [10]. Movement disability was additionally included because physical limitations may restrict peer interaction, participation in school activities, and school attendance [37]. The framework was specified to guide covariate adjustment rather than to assert a proven causal structure. Because SDQ and school-related outcomes were measured concurrently in 2023, the SDQ-to-outcome pathway was treated as exploratory. All multivariable regression and indirect-association models were adjusted for sex, age, BMI, only-child status, exercise habit, good sleep, hospitalization history, parental scolding, and movement disability.

### 2.5. Statistical Analysis

No a priori sample size calculation was performed because this was a secondary analysis of an existing community-based cohort; all eligible participants with the required data were included. School refusal tendency occurred in 84 of 314 participants. The 84 school refusal tendency events corresponded to approximately 7.6 events per predictor parameter in the primary fully adjusted Model 3. The full baseline-SDQ-adjusted Model 3 contained 12 predictor parameters, corresponding to approximately 7.0 events per predictor parameter. Because these event-to-parameter ratios may limit precision for small associations and sparsely distributed covariates, model stability was evaluated in additional sensitivity analyses.

Continuous variables are presented as means and standard deviations (SDs), and categorical variables are presented as numbers and percentages. Participant characteristics were compared according to school refusal tendency in 2023. Welch’s *t*-tests were used for continuous variables, and chi-square tests or Fisher’s exact tests were used for categorical variables, as appropriate.

To assess potential attrition bias, baseline characteristics were compared between participants included in the final analysis (*n* = 314) and eligible participants not included in the final analytical sample (*n* = 23), comprising 15 participants lost to follow-up and 8 participants with missing data on key analytical variables. Age, BMI, and ISI total score were compared using Welch’s *t*-tests. Sex, only-child status, exercise habit, good sleep, hospitalization history, parental scolding, and movement disability were compared using two-sided Fisher’s exact tests.

The primary exposure was the ISI total score measured in 2020. The SDQ total difficulties score measured in 2023 was treated as the candidate intermediate variable in the exploratory indirect-association analysis. The primary outcome was school refusal tendency measured in 2023. Frequent school absence in 2023 was examined as a supplementary outcome because of its low event count. Baseline SDQ scores from 2020 were used in sensitivity analyses. Other baseline covariates included sex, age, BMI, only-child status, exercise habit, good sleep, hospitalization history, parental scolding, and movement disability.

First, multivariable linear regression models were used to examine the association between ISI total score in 2020 and SDQ scores in 2023. Separate models were fitted for SDQ total difficulties, emotional symptoms, conduct problems, hyperactivity/inattention, peer problems, prosocial behavior, internalizing problems, and externalizing problems. Regression coefficients (β), standard errors, 95% confidence intervals (CIs), and *p*-values were reported.

Second, multivariable logistic regression models were used to examine associations with school refusal tendency in 2023. Three models were fitted: Model 1 included ISI total score and covariates; Model 2 included SDQ total difficulties and covariates; and Model 3 included both ISI total score and SDQ total difficulties with covariates. Adjusted odds ratios (ORs), 95% CIs, and *p*-values were reported.

Third, exploratory statistical indirect-association analyses were conducted to examine the association between ISI total score in 2020 and school outcomes in 2023 through SDQ total difficulties in 2023. Path a, representing the association between ISI and SDQ total difficulties, was estimated using linear regression. Paths b, c, and c′, representing the association between SDQ and the school outcome, the total association of ISI with the school outcome, and the direct association of ISI after adjustment for SDQ, respectively, were estimated using logistic regression for school refusal tendency. Because frequent school absence was rare, Firth bias-reduced logistic regression was used for models with frequent school absence as the outcome. Indirect effects were estimated on the log-odds scale using the Monte Carlo product-of-coefficients method with 95% CIs [38,39]. The analysis was based on separately fitted regression models and was neither a latent-variable structural equation model nor a causal mediation analysis.

Supplementary indirect-association analyses were conducted using SDQ subdomain scores as candidate intermediate variables, including emotional symptoms, conduct problems, hyperactivity/inattention, peer problems, prosocial behavior, internalizing problems, and externalizing problems. Each SDQ subdomain was evaluated in a separate model. All indirect-association models were adjusted for sex, age, BMI, only-child status, exercise habit, good sleep, hospitalization history, parental scolding, and movement disability.

Sensitivity analyses were conducted to evaluate the stability of the school refusal tendency models. First, Models 1–3 and the core indirect-effect model were re-estimated after excluding movement disability from the adjustment set because only three participants had this characteristic. Second, the school refusal tendency models were re-estimated using Firth bias-reduced logistic regression. Third, a parsimonious adjustment set comprising sex, age, BMI, exercise habit, and only-child status was defined on theoretical grounds and applied to Models 1–3 and the indirect-effect analysis; this set was not selected according to statistical significance. The distribution of the ISI total score was additionally examined using skewness, the proportions scoring 18 and 17–18, a quadratic ISI term, a restricted cubic spline with three degrees of freedom, and a quadratic Firth logistic model. Likelihood-ratio tests were used to compare the quadratic and spline specifications with the linear ISI model. These distributional and nonlinearity analyses were considered exploratory.

As an additional sensitivity analysis addressing pre-existing psychological and behavioral difficulties, all core models were re-estimated with adjustment for baseline SDQ total difficulties. Path a was estimated by regressing follow-up SDQ total difficulties on baseline ISI, baseline SDQ total difficulties, and the prespecified covariates. Baseline-SDQ-adjusted Models 1–3 were then fitted for school refusal tendency, and the corresponding indirect effect was recalculated using 20,000 Monte Carlo draws. These analyses were repeated using Firth bias-reduced logistic regression and the theoretically defined parsimonious adjustment set. Each follow-up SDQ domain was also adjusted for its corresponding baseline domain in domain-specific indirect-association analyses. Baseline-SDQ-adjusted Firth models were fitted for frequent school absence. As a secondary robustness check, change in SDQ total difficulties (follow-up minus baseline) was regressed on baseline ISI and the full covariate set. Variance inflation factors were examined for the full baseline-SDQ-adjusted Model 3.

Because the candidate intermediate variable and outcomes were both measured in 2023, the indirect-association analyses were interpreted as exploratory and non-causal. All statistical tests were two-sided, and *p*-values < 0.05 were considered statistically significant. All analyses were conducted using R version 4.4.2 (R Foundation for Statistical Computing, Vienna, Austria). Data management and descriptive analyses were performed using the readxl (version 1.4.3), dplyr (version 1.1.4), tidyr (version 1.3.1), and tableone (version 0.13.2) packages. Linear regression and standard logistic regression models were fitted using the stats package (version 4.4.2). Firth bias-reduced logistic regression models were fitted using the logistf package (version 1.26.0). Monte Carlo confidence intervals for indirect effects were estimated using the product-of-coefficients approach with 20,000 simulations. A fixed random seed was set to ensure reproducibility of the Monte Carlo procedures. The statistical analysis code is available from the corresponding author upon reasonable request. A complete-case approach was used for the primary analyses.

## 3. Results

### 3.1. Participant Characteristics

The final analytical sample included 314 children and adolescents. Among them, 84 participants (26.8%) showed school refusal tendency in 2023, whereas 230 participants (73.2%) did not. Frequent school absence was observed in 16 participants (5.1%).

Baseline characteristics of participants included in the final analysis and eligible participants not included in the final analytical sample are presented in Supplementary Table S2. No statistically significant differences were observed in sex, age, BMI, baseline ISI total score, only-child status, exercise habit, good sleep, hospitalization history, parental scolding, or movement disability between the two groups (all *p* > 0.05). However, the absolute standardized mean differences for exercise habit and parental scolding indicated that some descriptive imbalance remained possible in the small non-retained group.

Table 1 presents the participant characteristics according to school refusal tendency. The proportion of children with an exercise habit was significantly lower among those with school refusal tendency than among those without school refusal tendency (75.0% vs. 95.2%, *p* < 0.001). Frequent school absence was more common among children with school refusal tendency than among those without school refusal tendency (15.5% vs. 1.3%, *p* < 0.001). Only-child status tended to be less common in the school refusal group, although the difference did not reach statistical significance (10.7% vs. 20.0%, *p* = 0.055). No significant differences were observed for sex, age, BMI, good sleep, hospitalization history, parental scolding, or movement disability.

The total ISI score in 2020 was significantly lower among children with school refusal tendency than among those without school refusal tendency (14.51 ± 3.30 vs. 15.39 ± 1.71, *p* = 0.023). Among ISI subdomains, the school refusal group showed significantly lower independence scores (3.38 ± 1.16 vs. 3.80 ± 0.56, *p* = 0.002) and safety/trust scores (1.73 ± 0.61 vs. 1.96 ± 0.27, *p* < 0.001). Participation scores also tended to be lower in the school refusal group, although the difference did not reach statistical significance (2.89 ± 0.99 vs. 3.12 ± 0.83, *p* = 0.062). No significant differences were observed for interaction or social curiosity scores.

Regarding SDQ scores in 2023, children with school refusal tendency had significantly higher SDQ total difficulties than those without school refusal tendency (11.20 ± 5.22 vs. 8.30 ± 4.11, *p* < 0.001). The school refusal group also had higher emotional symptoms (2.54 ± 1.81 vs. 1.39 ± 1.57, *p* < 0.001), peer problems (2.86 ± 1.66 vs. 1.76 ± 1.46, *p* < 0.001), and internalizing problems (5.39 ± 2.87 vs. 3.14 ± 2.43, *p* < 0.001). Prosocial behavior was significantly lower in the school refusal group than in the no school refusal group (4.63 ± 1.25 vs. 5.54 ± 1.98, *p* < 0.001). Conduct problems, hyperactivity/inattention, and externalizing problems were higher in the school refusal group, but these differences were not statistically significant.

### 3.2. Association Between Baseline Social Interaction and Follow-Up SDQ Scores

Table 2 shows the adjusted linear regression results for the association between baseline ISI total score in 2020 and SDQ scores in 2023. After adjustment for sex, age, BMI, only-child status, exercise habit, good sleep, hospitalization history, parental scolding, and movement disability, higher ISI total score in 2020 was significantly associated with lower SDQ total difficulties in 2023 (β = −0.830, 95% CI: −1.033 to −0.627, *p* < 0.001). This indicates that each 1-point increase in ISI total score was associated with an average 0.83-point decrease in SDQ total difficulties at follow-up.

Higher baseline ISI was also significantly associated with lower scores for emotional symptoms (β = −0.212, 95% CI: −0.294 to −0.130, *p* < 0.001), conduct problems (β = −0.181, 95% CI: −0.245 to −0.118, *p* < 0.001), hyperactivity/inattention (β = −0.285, 95% CI: −0.377 to −0.193, *p* < 0.001), and peer problems (β = −0.152, 95% CI: −0.229 to −0.074, *p* < 0.001). In addition, higher ISI was significantly associated with lower internalizing problems (β = −0.364, 95% CI: −0.492 to −0.236, *p* < 0.001) and externalizing problems (β = −0.466, 95% CI: −0.596 to −0.337, *p* < 0.001). Conversely, higher ISI was associated with higher prosocial behavior scores (β = 0.118, 95% CI: 0.026 to 0.210, *p* = 0.012).

### 3.3. Adjusted Associations with School Refusal Tendency

Table 3 presents the adjusted logistic regression models for school refusal tendency in 2023. In Model 1, which included ISI total score and covariates, higher ISI total score in 2020 was significantly associated with lower odds of school refusal tendency in 2023 (OR = 0.832, 95% CI: 0.741 to 0.934, *p* = 0.002). This suggests that each 1-point increase in baseline ISI total score was associated with approximately 16.8% lower odds of later school refusal tendency.

In Model 2, which included SDQ total difficulties and covariates, higher SDQ total difficulties in 2023 were significantly associated with increased odds of school refusal tendency in 2023 (OR = 1.175, 95% CI: 1.098 to 1.258, *p* < 0.001). Each 1-point increase in SDQ total difficulties was associated with approximately 17.5% higher odds of school refusal tendency.

In Model 3, both ISI total score and SDQ total difficulties were included simultaneously with covariates. SDQ total difficulties remained significantly associated with school refusal tendency (OR = 1.155, 95% CI: 1.073 to 1.244, *p* < 0.001), whereas the direct association between ISI total score and school refusal tendency was no longer statistically significant after adjustment for SDQ total difficulties (OR = 0.925, 95% CI: 0.811 to 1.056, *p* = 0.250). Among covariates, exercise habit was consistently associated with lower odds of school refusal tendency across all models.

### 3.4. Core Exploratory Indirect-Association Analysis

Table 4 presents the exploratory indirect-association analysis using SDQ total difficulties in 2023 as the intermediate variable between ISI total score in 2020 and school outcomes in 2023. For the primary outcome of school refusal tendency, the association between ISI total score and SDQ total difficulties was significant (path a: β = −0.830, 95% CI: −1.033 to −0.627). The association between SDQ total difficulties and school refusal tendency, adjusted for ISI and covariates, was also significant (path b: OR = 1.155, 95% CI: 1.073 to 1.244). The total association of ISI with school refusal tendency was statistically significant (OR = 0.832, 95% CI: 0.741 to 0.934), whereas the direct association after adjustment for SDQ total difficulties was not statistically significant (OR = 0.925, 95% CI: 0.811 to 1.056). The indirect effect through SDQ total difficulties was statistically significant on the log-odds scale (indirect effect = −0.120, 95% CI: −0.193 to −0.055).

**Figure 3:**
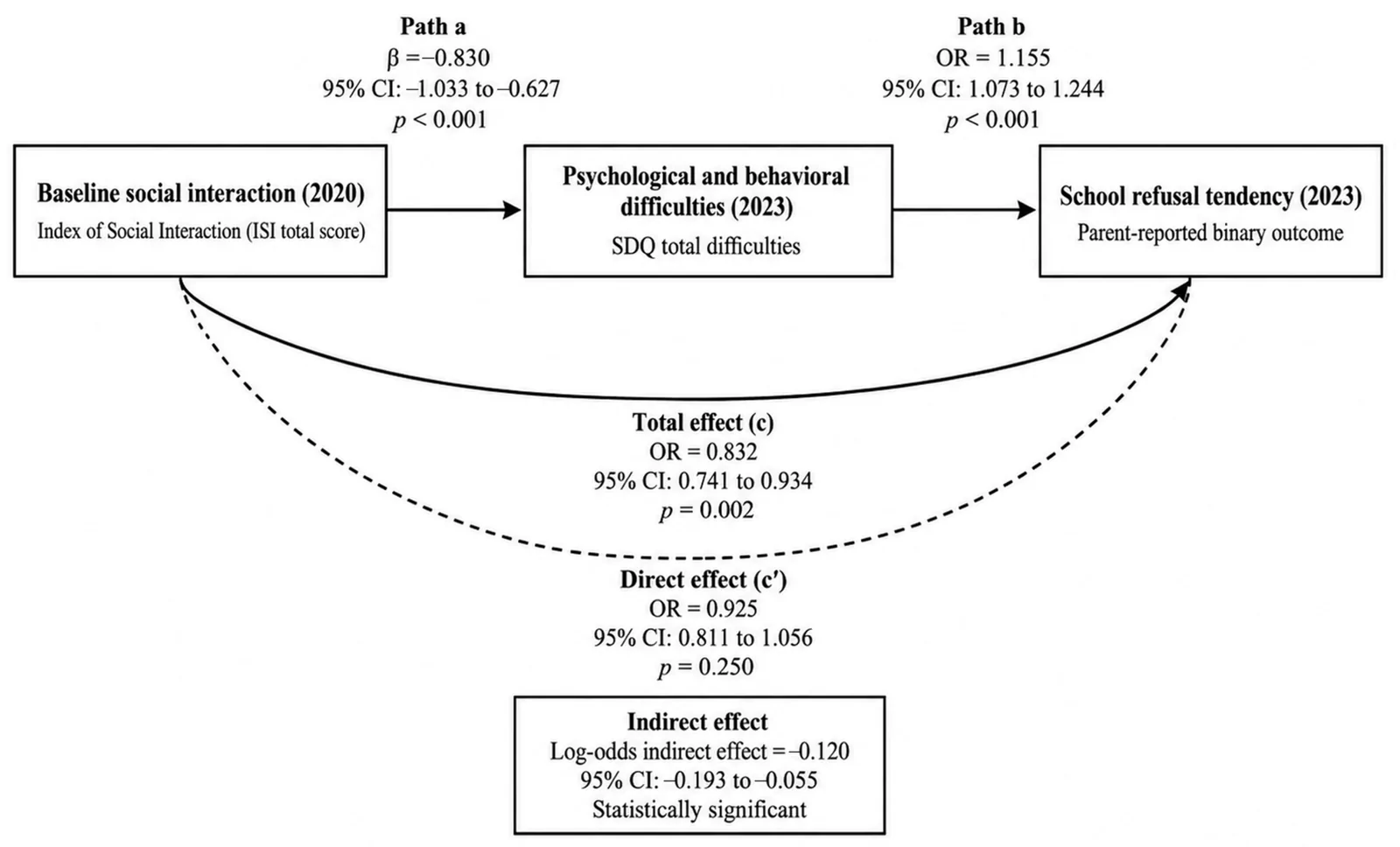
Exploratory statistical indirect-association model of baseline social interaction, SDQ total difficulties, and school refusal tendency. Note. Path a was estimated using linear regression. Paths b, c, and c′ were estimated using logistic regression. The indirect effect is reported on the log-odds scale using Monte Carlo product-of-coefficients confidence intervals. All models were adjusted for sex, age, BMI, only-child status, exercise habit, good sleep, hospitalization history, parental scolding, and movement disability. ISI, Index of Social Interaction; SDQ, Strengths and Difficulties Questionnaire; OR, odds ratio; CI, confidence interval.

These findings were consistent with a significant exploratory statistical indirect association linking higher baseline social interaction with lower odds of later school refusal tendency through lower psychological and behavioral difficulties. Specifically, higher social interaction in 2020 was associated with lower SDQ total difficulties in 2023, which were in turn associated with lower odds of school refusal tendency in 2023. Because SDQ and school refusal tendency were measured at the same follow-up wave, this pattern should not be interpreted as evidence of temporal or causal mediation (Figure 3).

Frequent school absence was examined as a supplementary outcome because of its low event count. In the Firth logistic regression model, higher SDQ total difficulties were associated with higher odds of frequent school absence (OR = 1.381, 95% CI: 1.159 to 1.645). The total effect of ISI on frequent school absence was significant (OR = 0.640, 95% CI: 0.534 to 0.768), whereas the direct effect after adjustment for SDQ total difficulties was not statistically significant (OR = 0.830, 95% CI: 0.658 to 1.047). The indirect effect was significant (indirect effect = −0.269, 95% CI: −0.439 to −0.119). However, because only 16 participants experienced frequent school absence, this result should be interpreted as supplementary and exploratory.

### 3.5. Supplementary Indirect-Association Analyses Using SDQ Subdomains

Supplementary Table S1 presents exploratory indirect-association analyses using SDQ subdomain scores as candidate intermediate variables. For school refusal tendency, significant indirect effects were observed for emotional symptoms, peer problems, prosocial behavior, and internalizing problems. Specifically, emotional symptoms showed a significant indirect effect in the association between ISI and school refusal tendency (indirect effect = −0.079, 95% CI: −0.130 to −0.036). Peer problems also showed a significant indirect effect (indirect effect = −0.062, 95% CI: −0.110 to −0.025). Prosocial behavior showed a significant indirect effect in the expected direction (indirect effect = −0.026, 95% CI: −0.060 to −0.002), indicating that higher baseline ISI was associated with higher prosocial behavior, which was in turn associated with lower odds of school refusal tendency. Internalizing problems showed the strongest subdomain-level indirect effect for school refusal tendency (indirect effect = −0.116, 95% CI: −0.181 to −0.062).

In contrast, conduct problems, hyperactivity/inattention, and externalizing problems did not show significant indirect effects for school refusal tendency. These findings suggest that the exploratory statistical indirect association between baseline social interaction and later school refusal tendency was more strongly related to internalizing difficulties, particularly emotional symptoms and peer problems, than to externalizing difficulties.

For frequent school absence, significant indirect effects were observed for emotional symptoms, conduct problems, hyperactivity/inattention, peer problems, internalizing problems, and externalizing problems. Emotional symptoms showed a significant indirect effect (indirect effect = −0.099, 95% CI: −0.187 to −0.027), as did conduct problems (indirect effect = −0.078, 95% CI: −0.169 to −0.001), hyperactivity/inattention (indirect effect = −0.094, 95% CI: −0.188 to −0.011), peer problems (indirect effect = −0.058, 95% CI: −0.132 to −0.002), internalizing problems (indirect effect = −0.125, 95% CI: −0.231 to −0.038), and externalizing problems (indirect effect = −0.121, 95% CI: −0.233 to −0.024). Prosocial behavior did not show a significant indirect effect for frequent school absence. However, these findings should be interpreted cautiously because frequent school absence was rare in the present sample.

### 3.6. Sensitivity and Nonlinearity Analyses

The ISI total score had a mean of 15.15 (SD = 2.28), a median of 16, and a negatively skewed distribution (skewness = −1.842). Twenty-one participants (6.7%) scored 18, and 86 (27.4%) scored 17–18. Exploratory nonlinearity analyses suggested curvature in the ISI–school refusal tendency association: the quadratic ISI term was significant in ordinary and Firth logistic regression models (both *p* < 0.001), and quadratic and restricted cubic spline models improved fit compared with the linear ISI model (both model-comparison *p* < 0.001; Supplementary Table S4). Because observations at the lower end of the ISI distribution were sparse, these findings should be interpreted cautiously.

The exploratory indirect effect through SDQ total difficulties also remained statistically significant after exclusion of movement disability (−0.121, 95% CI: −0.194 to −0.055), in the parsimonious model (−0.113, 95% CI: −0.186 to −0.048), and when Firth logistic regression was used for the outcome model (−0.112, 95% CI: −0.182 to −0.049).

Sensitivity analyses yielded materially similar estimates after exclusion of movement disability, in parsimonious models adjusted for sex, age, BMI, exercise habit, and only-child status, and using Firth bias-reduced logistic regression (Supplementary Table S3). In Model 1, the OR for ISI was 0.831 (95% CI: 0.742–0.932; *p* = 0.002) after exclusion of movement disability, 0.845 (95% CI: 0.759–0.942; *p* = 0.002) in the parsimonious model, and 0.841 (95% CI: 0.750–0.939; *p* = 0.002) in the Firth model. In Model 3, the direct ISI estimate remained non-significant and the SDQ estimate remained significant in all sensitivity models.

### 3.7. Baseline SDQ-Adjusted Sensitivity Analyses

Baseline SDQ data were complete for all 314 participants. Baseline SDQ total difficulties averaged 10.15 ± 5.34 and were moderately correlated with follow-up SDQ total difficulties (r = 0.568). In the full baseline-SDQ-adjusted model, higher baseline ISI remained associated with lower follow-up SDQ total difficulties (path a: β = −0.554, 95% CI: −0.741 to −0.367; *p* < 0.001). The baseline-SDQ-adjusted total association between ISI and school refusal tendency was also significant in standard logistic regression (OR = 0.876, 95% CI: 0.775–0.991; *p* = 0.035).

In baseline-SDQ-adjusted Model 3, follow-up SDQ total difficulties remained associated with school refusal tendency (OR = 1.129, 95% CI: 1.039–1.225; *p* = 0.004), whereas the direct ISI association was not statistically significant (OR = 0.931, 95% CI: 0.816–1.063; *p* = 0.293). The indirect effect was attenuated compared with the primary analysis but remained statistically significant (−0.0670, 95% CI: −0.1235 to −0.0200). Corresponding Firth (−0.0617, 95% CI: −0.1163 to −0.0160) and parsimonious-model estimates (−0.0614, 95% CI: −0.1179 to −0.0128) were similar (Supplementary Tables S5 and S6). The maximum variance inflation factor was 1.82, indicating no meaningful multicollinearity.

After adjustment for the corresponding baseline SDQ domain, indirect effects for school refusal tendency remained statistically significant through emotional symptoms (−0.0526, 95% CI: −0.0973 to −0.0176), peer problems (−0.0341, 95% CI: −0.0712 to −0.0068), and internalizing problems (−0.0694, 95% CI: −0.1210 to −0.0288), but not through conduct problems, hyperactivity/inattention, prosocial behavior, or externalizing problems (Supplementary Table S7).

For frequent school absence, the baseline-SDQ-adjusted Firth indirect effect remained significant (−0.1350, 95% CI: −0.2675 to −0.0229), although this result remains supplementary because only 16 events occurred (Supplementary Table S8). The association between baseline ISI and the simple SDQ change score was not statistically significant (β = −0.110, 95% CI: −0.347 to 0.128; *p* = 0.365; Supplementary Table S9).

## 4. Discussion

### 4.1. Principal Findings

This three-year longitudinal community-based study examined whether baseline multidimensional social interaction was associated with later school refusal tendency among elementary and junior high school students aged 12 years or older, and whether an exploratory statistical indirect association was observed through psychological and behavioral difficulties. Higher baseline ISI was associated with lower follow-up SDQ difficulties and lower odds of school refusal tendency in the primary analyses. The direct ISI association was no longer statistically significant after inclusion of follow-up SDQ total difficulties, whereas the exploratory indirect effect was significant. Importantly, adjustment for baseline SDQ total difficulties attenuated but did not eliminate this pattern: baseline ISI remained associated with lower follow-up SDQ difficulties, and the indirect effect remained significant in the full standard, Firth bias-reduced, and parsimonious models. Baseline-domain-adjusted analyses retained indirect effects through emotional symptoms, peer problems, and internalizing problems. The baseline-SDQ-adjusted indirect effect for frequent school absence also remained significant, although this result requires particular caution because only 16 events occurred.

### 4.2. Interpretation in Relation to Previous Research

The finding that higher social interaction was associated with lower later psychological and behavioral difficulties is consistent with the broader child development literature emphasizing the role of interpersonal relationships, participation, and social competence in children’s mental health and school adjustment [16,21,23]. Social interaction may provide opportunities for emotional regulation, peer learning, help-seeking, and the development of a sense of belonging, all of which are closely related to children’s mental health and school connectedness [40,41]. In contrast, limited social interaction may reduce exposure to supportive relationships and adaptive social learning experiences, thereby increasing vulnerability to emotional symptoms and peer-related difficulties [22,42]. Although the ISI was originally developed as a community-based measure of social relatedness and has been used mainly in adult and older-adult community health research, it was designed for community-dwelling populations aged 12 years and older [24,26]. Therefore, its multidimensional framework may be useful for capturing broader social engagement among adolescents in community settings.

Adolescent-specific measures differ from the ISI in both scope and setting. The Psychological Sense of School Membership scale focuses on students’ perceived acceptance, inclusion, respect, and belonging within the school environment [43], whereas the Child and Adolescent Social Support Scale assesses perceived support from sources such as parents, teachers, classmates, and close friends [44]. A scoping review identified a wide range of adolescent connectedness measures, most of which target domain-specific school, family, peer, or community connectedness [45]. In contrast, the ISI combines interpersonal interaction and participation with social curiosity, independence, and safety/trust across everyday community life. This broader scope may help capture upstream community engagement beyond the school setting, but it is less specific to school belonging or perceived social support and should not be considered a substitute for adolescent-validated school-connectedness or social-support instruments [40,43,44,45]. Future studies should administer the ISI alongside such measures to evaluate convergent and incremental validity among adolescents.

The descriptive findings for the ISI subdomains provide additional insight. Children with school refusal tendency had significantly lower total ISI scores than those without school refusal tendency. At the subdomain level, they showed significantly lower independence and safety/trust scores, while participation scores showed a non-significant tendency to be lower. These results should be interpreted cautiously because the subdomain analyses were descriptive. Nevertheless, they suggest that aspects of social engagement related to daily independence and perceived availability of support may be particularly relevant to school adaptation. Independence may reflect proactive daily engagement and motivation for active living, whereas safety/trust may reflect whether children perceive that they have reliable people or systems to consult in difficult situations. Such perceptions may influence children’s willingness to attend school, seek help, and remain connected to school or community life [46,47].

The exploratory nonlinearity analyses further suggested that the association between ISI scores and school refusal tendency may not be uniform across the full score range. Although the primary linear model estimated an average inverse association, the quadratic and spline models indicated curvature, and school refusal tendency was not monotonically lower at every successively higher ISI score. This pattern is broadly consistent with evidence that the psychological benefits of increasing social-interaction quantity diminish after moderate levels are reached rather than increasing indefinitely [48]. Experience-sampling research has also shown that simply being with others does not necessarily alleviate loneliness and that the quality and subjective meaning of social contact may be more important than contact alone [49]. Accordingly, a high ISI score should not be interpreted as indicating that every form or level of social engagement is necessarily beneficial. However, the ISI assesses multidimensional engagement rather than interaction quantity alone, and its distribution in this sample was concentrated toward the upper end with relatively few low-score observations. The estimated curve should therefore be regarded as hypothesis-generating rather than definitive.

The strong association between SDQ total difficulties and school refusal tendency is also consistent with previous research showing that psychological distress, anxiety, depressive symptoms, and peer difficulties are closely related to school refusal and school attendance problems [1,7,9]. In the present study, the school refusal group had higher SDQ total difficulties, emotional symptoms, peer problems, and internalizing problems, as well as lower prosocial behavior. In contrast, conduct problems, hyperactivity/inattention, and externalizing problems were not significantly different between groups in the descriptive analyses and did not show significant indirect effects for school refusal tendency. This pattern suggests that school refusal tendency in this community sample may be more closely related to internalizing and interpersonal difficulties than to overt behavioral or externalizing problems.

The timing of the study also requires consideration. The baseline survey began in April 2020, during the initial period of COVID-19-related school disruption in Japan. A regression-discontinuity study found that the nationwide elementary-school closures were associated with changes in children’s health-related behavior and increased maternal anxiety about child-rearing [50]. A Japanese survey of families with children aged 6–15 years further found that full school closure was associated with greater child and parent mental health problems, while partial closure was associated with higher anxiety symptoms [51]. In addition, Japanese public schools varied in their capacity to provide online education, partly because of differences in available information and communication technology resources [52]. These disruptions may have affected social interaction, psychological and behavioral difficulties, and school adaptation during follow-up. Because individual exposure to closures, remote education, and pandemic-related stress was not measured, their influence cannot be separated from the observed associations.

The findings should also be considered within the Japanese school context. Research on ijime in Japanese middle schools has described complex classroom and peer-group dynamics, including relational exclusion, social manipulation, and the further isolation of students who lack trusting peer relationships [53]. Research with Japanese junior high school students has also linked roles in ijime situations with differing tendencies toward conformity to the majority [54], while sociocultural analyses have emphasized the group-based and relational features of ijime [55]. These contextual features may make peer belonging, trusted relationships, and perceived safety particularly relevant to students’ willingness to remain engaged with school. However, bullying, conformity, classroom climate, and school belonging were not directly measured in the present study; these mechanisms therefore represent contextual interpretations rather than explanations established by the data.

### 4.3. Exploratory Statistical Indirect Association and Possible Psychosocial Pathways

The primary exploratory analysis identified a statistically significant indirect association linking baseline social interaction with later school refusal tendency through follow-up psychological and behavioral difficulties. Adjustment for baseline SDQ total difficulties reduced the magnitude of path a and the indirect effect, indicating that pre-existing psychological and behavioral difficulties accounted for part of the original association. Nevertheless, baseline ISI remained significantly associated with follow-up SDQ total difficulties, and the baseline-SDQ-adjusted indirect effect remained statistically significant across standard logistic, Firth bias-reduced, and parsimonious models. The total ISI association remained significant in the full standard and Firth models but was attenuated to marginal non-significance in the parsimonious model, while the direct ISI estimate remained non-significant in all baseline-adjusted Model 3 specifications. These results strengthen evidence that the findings were not solely attributable to baseline psychological and behavioral difficulties, while also showing that estimates were sensitive in magnitude to adjustment for prior SDQ levels.

Domain-specific analyses further characterized this pattern. In the original analyses, emotional symptoms, peer problems, prosocial behavior, and internalizing problems showed significant indirect effects for school refusal tendency. After adjustment for the corresponding baseline domain, indirect effects remained for emotional symptoms, peer problems, and internalizing problems, whereas the prosocial-behavior indirect effect was no longer statistically significant. This pattern is consistent with evidence that school absenteeism and attendance problems are closely related to child mental health, particularly internalizing symptoms [56]. Reluctance to attend school may be associated with anxiety, emotional distress, peer relationship problems, and difficulties in feeling accepted or connected within the school environment [9,57]. Higher social interaction may be related to greater peer connection, social confidence, and access to trusted interpersonal support [47,58], whereas weaker social engagement may be associated with social withdrawal or interpersonal vulnerability [1]. These mechanisms were not directly tested.

For frequent school absence, the baseline-SDQ-adjusted Firth analysis also retained a statistically significant indirect effect through follow-up SDQ total difficulties. However, because only 16 participants experienced frequent school absence, this result should remain supplementary and exploratory. More importantly, baseline SDQ adjustment does not establish temporal ordering between follow-up SDQ difficulties and school outcomes because both were measured in 2023. The findings therefore remain evidence of statistical indirect association rather than a causal mediating process, and confirmation requires designs in which the candidate intermediate variable is assessed before the school-related outcome.

### 4.4. Practical Implications

These findings may help clinicians, school nurses, educators, and policymakers recognize multidimensional social engagement, emotional symptoms, and peer difficulties as domains that may warrant further assessment and support. However, the present study does not validate the ISI or the single school-refusal item as screening instruments, and routine screening cannot be recommended on the basis of these findings alone. School- and community-based initiatives that provide opportunities for safe participation, peer connection, trusted adult support, and help-seeking may nevertheless be relevant for students who report reluctance to attend school.

The consistent association between exercise habit and lower odds of school refusal tendency across logistic regression models also warrants attention. Exercise habit was included as a covariate rather than specified as a primary exposure, so this result should not be interpreted as evidence of a causal protective effect. Nevertheless, a systematic review and meta-analysis of 38 studies reported a small positive association between physical activity and behavioral, emotional, and cognitive dimensions of school engagement [59]. Studies of middle school students have further suggested that interpersonal relationships, academic support, perceived social support, and learning engagement may statistically link physical activity with academic engagement or achievement [60,61]. Experimental evidence also indicates that physically active academic lessons may improve children’s classroom time-on-task [62]. Together, these findings suggest that exercise habit may reflect not only physical activity itself but also structured daily routines, opportunities for peer interaction, perceived support, and behavioral self-regulation. These mechanisms were not directly assessed in the present study and should be examined in future longitudinal research.

### 4.5. Strengths and Limitations

The strengths of this study include its longitudinal community-based design, the use of baseline social interaction data and follow-up psychological and school-related outcomes, and adjustment for multiple baseline covariates. The study used the parent-reported Japanese version of the SDQ at baseline and follow-up and examined total difficulties and subdomain-level patterns. Sensitivity analyses controlled for baseline SDQ total difficulties or the corresponding baseline domain, thereby reducing concern that the findings solely reflected pre-existing psychological and behavioral difficulties. Model stability was also evaluated by excluding movement disability, applying Firth bias-reduced logistic regression, using a parsimonious theoretically defined adjustment set, and assessing ISI nonlinearity.

Several limitations should also be acknowledged. First, school refusal tendency and frequent school absence were assessed using single parent- or guardian-reported questionnaire items rather than clinical diagnoses or administrative attendance records. The school absence item did not distinguish refusal-related absence from absence due to illness, family circumstances, or other reasons. Second, follow-up SDQ scores and school-related outcomes were measured at the same wave in 2023 and by the same reporter; shared method variance may therefore have strengthened their observed association, and temporal ordering between the candidate intermediate variable and outcomes could not be established. Baseline SDQ adjustment reduces prior-state confounding but does not resolve this temporal limitation. Third, baseline school refusal tendency was unavailable, so pre-existing reluctance to attend school and reverse or residual confounding could not be directly evaluated. Fourth, although the ISI is intended for community populations aged 12 years or older and showed acceptable internal consistency in this sample (Cronbach’s α = 0.757), adolescent-specific construct-validity evidence remains limited; the total score should therefore be interpreted as a broad multidimensional indicator of social interaction and engagement. Fifth, the study was conducted in a village in Aichi Prefecture, central Japan, which may limit generalizability to urban areas or other cultural settings. Sixth, although the attrition analysis found no statistically significant baseline differences between retained and non-retained participants, the non-retained group was small, descriptive imbalances were observed for some variables, and selection bias cannot be excluded. Seventh, no a priori sample size calculation was performed. The primary fully adjusted Model 3 had approximately 7.6 events per predictor parameter, and the baseline-SDQ-adjusted full Model 3 had approximately 7.0. Although below the traditional rule of thumb of 10 events per predictor parameter, the principal indirect-effect estimates remained statistically significant in Firth bias-reduced and parsimonious baseline-SDQ-adjusted analyses. Nevertheless, limited power to detect small associations and imprecision for sparsely distributed covariates cannot be excluded. Eighth, domain-specific analyses were exploratory and were not adjusted for multiple comparisons. Ninth, the exploratory nonlinearity analyses were based on a negatively skewed ISI distribution with relatively few observations at low scores. Tenth, the 2020–2023 study period overlapped with social changes related to the COVID-19 pandemic. Finally, frequent school absence occurred in only 16 participants, and analyses for this outcome should be interpreted cautiously.

## 5. Conclusions

In conclusion, higher baseline multidimensional social interaction was associated with lower subsequent psychological and behavioral difficulties and lower odds of school refusal tendency among elementary and junior high school students aged 12 years or older. The primary analysis identified a significant exploratory statistical indirect association through follow-up SDQ total difficulties. Adjustment for baseline SDQ attenuated but did not eliminate the indirect effect, and corresponding Firth and parsimonious analyses yielded similar estimates. Baseline-domain-adjusted analyses most consistently supported indirect associations through emotional symptoms, peer problems, and internalizing problems. These findings highlight the potential relevance of social engagement and internalizing-related difficulties for understanding school refusal tendency. However, because follow-up SDQ and school refusal tendency were assessed concurrently, the results do not establish a causal mediating process and require confirmation in studies with temporally ordered assessments.

## Figures and Tables

**Table 1:** Participant characteristics according to school refusal tendency.

Variable	Total (*N* = 314)	No School Refusal (*n* = 230)	School Refusal (*n* = 84)	*p*-Value
Female sex, *n* (%)	169 (53.8)	125 (54.3)	44 (52.4)	0.757
Only child, *n* (%)	55 (17.5)	46 (20.0)	9 (10.7)	0.055
Exercise habit, *n* (%)	282 (89.8)	219 (95.2)	63 (75.0)	<0.001
Good sleep, *n* (%)	290 (92.4)	211 (91.7)	79 (94.0)	0.496
Hospitalization history, *n* (%)	25 (8.0)	20 (8.7)	5 (6.0)	0.427
Parental scolding, *n* (%)	278 (88.5)	205 (89.1)	73 (86.9)	0.584
Movement disability, *n* (%)	3 (1.0)	2 (0.9)	1 (1.2)	1.000
Frequent school absence, *n* (%)	16 (5.1)	3 (1.3)	13 (15.5)	<0.001
Age, years, mean ± SD	13.15 ± 1.29	13.20 ± 1.36	13.04 ± 1.06	0.276
BMI, mean ± SD	16.70 ± 2.06	16.71 ± 2.06	16.66 ± 2.06	0.850
ISI total score, mean ± SD	15.15 ± 2.28	15.39 ± 1.71	14.51 ± 3.30	0.023
ISI score, mean ± SD	3.60 ± 0.60	3.63 ± 0.55	3.51 ± 0.70	0.150
ISI participation score, mean ± SD	3.06 ± 0.88	3.12 ± 0.83	2.89 ± 0.99	0.062
ISI curiosity score, mean ± SD	2.90 ± 0.84	2.87 ± 0.81	3.00 ± 0.92	0.253
ISI independence score, mean ± SD	3.69 ± 0.79	3.80 ± 0.56	3.38 ± 1.16	0.002
ISI safety/trust score, mean ± SD	1.90 ± 0.40	1.96 ± 0.27	1.73 ± 0.61	<0.001
SDQ total difficulties, mean ± SD	9.07 ± 4.61	8.30 ± 4.11	11.20 ± 5.22	<0.001
SDQ emotional symptoms, mean ± SD	1.69 ± 1.71	1.39 ± 1.57	2.54 ± 1.81	<0.001
SDQ conduct problems, mean ± SD	1.90 ± 1.34	1.83 ± 1.30	2.07 ± 1.45	0.190
SDQ hyperactivity/inattention, mean ± SD	3.43 ± 1.98	3.32 ± 1.88	3.74 ± 2.21	0.124
SDQ peer problems, mean ± SD	2.05 ± 1.59	1.76 ± 1.46	2.86 ± 1.66	<0.001
SDQ prosocial behavior, mean ± SD	5.30 ± 1.86	5.54 ± 1.98	4.63 ± 1.25	<0.001
SDQ internalizing problems, mean ± SD	3.75 ± 2.74	3.14 ± 2.43	5.39 ± 2.87	<0.001
SDQ externalizing problems, mean ± SD	5.33 ± 2.84	5.15 ± 2.69	5.81 ± 3.19	0.095

Note. Continuous variables are presented as mean ± SD; categorical variables are presented as *n* (%). *p*-values were calculated using Welch’s *t*-tests for continuous variables and chi-square or Fisher’s exact tests for categorical variables. School refusal tendency was measured in 2023; ISI and covariates were measured in 2020; SDQ scores were measured in 2023.

**Table 2:** Adjusted linear regression of baseline ISI with follow-up SDQ scores.

Outcome: SDQ Score in 2023	β for ISI Total Score 2020	SE	95% CI	*p*-Value
SDQ total difficulties	−0.830	0.103	−1.033, −0.627	<0.001
Emotional symptoms	−0.212	0.042	−0.294, −0.130	<0.001
Conduct problems	−0.181	0.032	−0.245, −0.118	<0.001
Hyperactivity/inattention	−0.285	0.047	−0.377, −0.193	<0.001
Peer problems	−0.152	0.039	−0.229, −0.074	<0.001
Prosocial behavior	0.118	0.047	0.026, 0.210	0.012
Internalizing problems	−0.364	0.065	−0.492, −0.236	<0.001
Externalizing problems	−0.466	0.066	−0.596, −0.337	<0.001

Note. β indicates the adjusted mean difference in SDQ score per 1-point increase in ISI total score. Models were adjusted for sex, age, BMI, only-child status, exercise habit, good sleep, hospitalization history, parental scolding, and movement disability.

**Table 3:** Logistic regression models for school refusal tendency.

Variable	Model 1 OR (95% CI), *p*	Model 2 OR (95% CI), *p*	Model 3 OR (95% CI), *p*
ISI total score	0.832 (0.741, 0.934), 0.002	—	0.925 (0.811, 1.056), 0.250
SDQ total difficulties	—	1.175 (1.098, 1.258), <0.001	1.155 (1.073, 1.244), <0.001
Female sex	0.828 (0.474, 1.447), 0.508	0.716 (0.404, 1.268), 0.252	0.747 (0.419, 1.333), 0.324
Age	0.925 (0.736, 1.164), 0.508	0.999 (0.790, 1.265), 0.996	0.996 (0.786, 1.261), 0.972
BMI	1.009 (0.878, 1.159), 0.903	1.026 (0.886, 1.187), 0.736	1.027 (0.886, 1.190), 0.723
Only child	0.558 (0.251, 1.238), 0.151	0.540 (0.234, 1.246), 0.149	0.544 (0.237, 1.250), 0.152
Exercise habit	0.142 (0.062, 0.326), <0.001	0.165 (0.071, 0.387), <0.001	0.162 (0.069, 0.380), <0.001
Good sleep	1.697 (0.547, 5.261), 0.360	1.973 (0.614, 6.338), 0.254	2.319 (0.681, 7.897), 0.178
Hospitalization history	0.694 (0.236, 2.042), 0.507	0.475 (0.153, 1.475), 0.198	0.467 (0.148, 1.471), 0.193
Parental scolding	0.951 (0.406, 2.224), 0.907	0.751 (0.313, 1.805), 0.522	0.786 (0.323, 1.909), 0.594
Movement disability	1.047 (0.067, 16.280), 0.974	0.438 (0.019, 9.852), 0.603	0.360 (0.013, 9.881), 0.545

Note. Outcome: school refusal tendency in 2023. ORs are adjusted odds ratios. Model 1 includes ISI and covariates; Model 2 includes SDQ total difficulties and covariates; Model 3 includes both ISI and SDQ total difficulties plus covariates.

**Table 4:** Core exploratory indirect-association analysis: ISI → SDQ total difficulties → school outcomes.

Outcome	Events, *n* (%)	Path a β (95% CI)	Path b OR (95% CI)	Total Effect c OR (95% CI)	Direct Effect c′ OR (95% CI)	Indirect Effect (95% CI)	Outcome Model
School refusal tendency	84 (26.8)	−0.830 (−1.033, −0.627)	1.155 (1.073, 1.244)	0.832 (0.741, 0.934)	0.925 (0.811, 1.056)	−0.120 (−0.193, −0.055)	Logistic regression
Frequent school absence	16 (5.1)	−0.830 (−1.033, −0.627)	1.381 (1.159, 1.645)	0.640 (0.534, 0.768)	0.830 (0.658, 1.047)	−0.269 (−0.439, −0.119)	Firth logistic

Note. Path a was estimated using linear regression. Paths b, c, and c′ were estimated using logistic regression for school refusal tendency and Firth logistic regression for frequent school absence. Indirect effects are reported on the log-odds scale using Monte Carlo product-of-coefficients confidence intervals. Frequent school absence had only 16 events and should be interpreted as supplementary exploratory evidence.

## Data Availability

The data presented in this study are available from the corresponding author upon reasonable request and with permission from the relevant ethics committee and local municipal authority. The data are not publicly available because of privacy and ethical restrictions related to research involving minors. The statistical analysis code is available from the corresponding author upon reasonable request.

## Supplementary Materials

The following supporting information can be downloaded at: https://www.mdpi.com/article/10.3390/children13081079/s1, Table S1. Exploratory indirect-association analyses using SDQ subdomain scores; Table S2. Comparison of baseline characteristics between participants included and not included in the final analytical sample; Table S3. Sensitivity analyses for school refusal tendency: (A) Logistic regression estimates; (B) Core indirect-effect sensitivity analyses; Table S4. Distribution, ceiling effect, and exploratory nonlinearity analyses of the ISI total score; Table S5. Baseline SDQ-adjusted sensitivity analyses for school refusal tendency; Table S6. Detailed baseline SDQ-adjusted logistic regression estimates; Table S7. Domain-specific indirect-association analyses adjusted for the corresponding baseline SDQ domain; Table S8. Baseline SDQ-adjusted Firth analyses for frequent school absence; Table S9. Data and model diagnostics for baseline SDQ-adjusted analyses.
