# Supplementary material for: Multidimensional Social Interaction and School Refusal Tendency Among Elementary and Junior High School Students: A Three-Year Longitudinal Study with an Exploratory Indirect-Association Analysis
Source: Children (Basel). 2026 Aug 14;13(8):1079. doi: 10.3390/children13081079 (PMC13510324; doi:10.3390/children13081079)
Supplement: Supplementary file 1 [file children-13-01079-s001.zip › children-4463172-supplementary.pdf]

Table S1. Exploratory indirect-association analyses using SDQ subdomain scores

Outcome	Candidate intermediate variable	Path a β (95% CI)	Path b OR (95% CI)	Total effect c OR (95% CI)	Direct effect c′ OR (95% CI)	Indirect effect (95% CI)	Significant indirect effect	Model
School refusal tendency	Emotional symptoms	-0.212 (-0.294, -0.130)	1.448 (1.221, 1.719)	0.832 (0.741, 0.934)	0.886 (0.780, 1.006)	-0.079 (-0.130, -0.036)	Yes	Logistic
School refusal tendency	Conduct problems	-0.181 (-0.245, -0.118)	1.075 (0.865, 1.335)	0.832 (0.741, 0.934)	0.843 (0.746, 0.953)	-0.013 (-0.056, 0.027)	No	Logistic
School refusal tendency	Hyperactivity/inattention	-0.285 (-0.377, -0.193)	1.043 (0.898, 1.213)	0.832 (0.741, 0.934)	0.841 (0.744, 0.951)	-0.012 (-0.057, 0.032)	No	Logistic
School refusal tendency	Peer problems	-0.152 (-0.229, -0.074)	1.510 (1.250, 1.823)	0.832 (0.741, 0.934)	0.869 (0.768, 0.983)	-0.062 (-0.110, -0.025)	Yes	Logistic
School refusal tendency	Prosocial behavior	0.118 (0.026, 0.210)	0.806 (0.682, 0.951)	0.832 (0.741, 0.934)	0.851 (0.754, 0.959)	-0.026 (-0.060, -0.002)	Yes	Logistic
School refusal tendency	Internalizing problems	-0.364 (-0.492, -0.236)	1.376 (1.221, 1.551)	0.832 (0.741, 0.934)	0.904 (0.792, 1.031)	-0.116 (-0.181, -0.062)	Yes	Logistic
School refusal tendency	Externalizing problems	-0.466 (-0.596, -0.337)	1.039 (0.935, 1.155)	0.832 (0.741, 0.934)	0.847 (0.747, 0.960)	-0.018 (-0.070, 0.031)	No	Logistic
Frequent school absence	Emotional symptoms	-0.212 (-0.294, -0.130)	1.590 (1.152, 2.197)	0.640 (0.534, 0.768)	0.727 (0.598, 0.882)	-0.099 (-0.187, -0.027)	Yes	Firth logistic
Frequent school absence	Conduct problems	-0.181 (-0.245, -0.118)	1.539 (0.997, 2.376)	0.640 (0.534, 0.768)	0.713 (0.582, 0.873)	-0.078 (-0.169, -0.001)	Yes	Firth logistic
Frequent school absence	Hyperactivity/inattention	-0.285 (-0.377, -0.193)	1.390 (1.044, 1.850)	0.640 (0.534, 0.768)	0.697 (0.569, 0.853)	-0.094 (-0.188, -0.011)	Yes	Firth logistic
Frequent school absence	Peer problems	-0.152 (-0.229, -0.074)	1.467 (1.012, 2.126)	0.640 (0.534, 0.768)	0.691 (0.572, 0.834)	-0.058 (-0.132, -0.002)	Yes	Firth logistic
Frequent school absence	Prosocial behavior	0.118 (0.026, 0.210)	0.794 (0.587, 1.074)	0.640 (0.534, 0.768)	0.672 (0.558, 0.808)	-0.027 (-0.079, 0.008)	No	Firth logistic
Frequent school absence	Internalizing problems	-0.364 (-0.492, -0.236)	1.411 (1.118, 1.781)	0.640 (0.534, 0.768)	0.748 (0.612, 0.916)	-0.125 (-0.231, -0.038)	Yes	Firth logistic
Frequent school absence	Externalizing problems	-0.466 (-0.596, -0.337)	1.295 (1.052, 1.594)	0.640 (0.534, 0.768)	0.730 (0.589, 0.905)	-0.121 (-0.233, -0.024)	Yes	Firth logistic

Note. Each SDQ subdomain was evaluated as a candidate intermediate variable in a separate exploratory indirect-association model. All models were adjusted for sex, age, BMI, only-child status, exercise habit, good sleep, hospitalization history, parental scolding, and movement disability. Indirect effects are reported on the log-odds scale using Monte Carlo product-of-coefficients confidence intervals. Frequent school absence models used Firth logistic regression because the event count was low. BMI, body mass index; CI, confidence interval; ISI, Index of Social Interaction; OR, odds ratio; SDQ, Strengths and Difficulties Questionnaire.

Table S2. Comparison of baseline characteristics between participants included and not included in the final analytical sample

Baseline characteristic	Final analysis (n = 314)	Not included (n = 23)	p-value	Statistical test	Absolute SMD
Age, years	13.15 ± 1.29	13.22 ± 1.57	0.849	Welch's t-test	0.049
BMI, kg/m²	16.70 ± 2.06	16.21 ± 1.57	0.169	Welch's t-test	0.241
ISI total score	15.15 ± 2.28	15.09 ± 2.19	0.891	Welch's t-test	0.029
Male sex, n (%)	145 (46.2)	12 (52.2)	0.667	Fisher's exact test	0.120
Only-child status, n (%)	55 (17.5)	5 (21.7)	0.577	Fisher's exact test	0.106
Exercise habit, n (%)	282 (89.8)	23 (100.0)	0.147	Fisher's exact test	0.463
Good sleep, n (%)	290 (92.4)	20 (87.0)	0.413	Fisher's exact test	0.177
Hospitalization history, n (%)	25 (8.0)	4 (17.4)	0.124	Fisher's exact test	0.283
Parental scolding, n (%)	278 (88.5)	23 (100.0)	0.152	Fisher's exact test	0.493
Movement disability, n (%)	3 (1.0)	0 (0.0)	1.000	Fisher's exact test	0.139

Note. Values are presented as mean ± SD or n (%). The group not included in the final analytical sample comprised 15 participants lost to follow-up and 8 participants with missing data on key analytical variables. Continuous variables were compared using Welch's t-tests; categorical variables were compared using two-sided Fisher's exact tests. Absolute SMDs are provided as descriptive measures of group imbalance. BMI, body mass index; ISI, Index of Social Interaction; SD, standard deviation; SMD, standardized mean difference.

Table S3. Sensitivity analyses for school refusal tendency

A. Logistic regression estimates

Analysis	Model	Predictor	OR	95% CI	p-value	Adjustment set	Interpretation
Primary standard logistic	Model 1	ISI	0.832	0.741–0.934	0.002	Full covariates including movement disability	OR < 1 indicates lower odds
Primary standard logistic	Model 2	SDQ	1.175	1.098–1.258	<0.001	Full covariates including movement disability	OR > 1 indicates higher odds
Primary standard logistic	Model 3	ISI	0.925	0.811–1.056	0.250	Full covariates including movement disability	Direct ISI estimate
Primary standard logistic	Model 3	SDQ	1.155	1.073–1.244	<0.001	Full covariates including movement disability	Adjusted SDQ estimate
Exclude movement disability	Model 1	ISI	0.831	0.742–0.932	0.002	Full covariates except movement disability	Materially unchanged
Exclude movement disability	Model 2	SDQ	1.171	1.095–1.252	<0.001	Full covariates except movement disability	Materially unchanged
Exclude movement disability	Model 3	ISI	0.928	0.814–1.059	0.269	Full covariates except movement disability	Remained non-significant
Exclude movement disability	Model 3	SDQ	1.151	1.070–1.239	<0.001	Full covariates except movement disability	Remained significant
Parsimonious core-confounder model	Model 1	ISI	0.845	0.759–0.942	0.002	Sex, age, BMI, exercise habit, only-child status	Materially unchanged
Parsimonious core-confounder model	Model 2	SDQ	1.149	1.080–1.223	<0.001	Sex, age, BMI, exercise habit, only-child status	Materially unchanged
Parsimonious core-confounder model	Model 3	ISI	0.944	0.832–1.071	0.371	Sex, age, BMI, exercise habit, only-child status	Remained non-significant
Parsimonious core-confounder model	Model 3	SDQ	1.132	1.056–1.215	<0.001	Sex, age, BMI, exercise habit, only-child status	Remained significant
Firth bias-reduced logistic	Model 1	ISI	0.841	0.750–0.939	0.002	Full covariates including movement disability	Materially unchanged
Firth bias-reduced logistic	Model 2	SDQ	1.163	1.091–1.245	<0.001	Full covariates including movement disability	Materially unchanged
Firth bias-reduced logistic	Model 3	ISI	0.933	0.820–1.061	0.289	Full covariates including movement disability	Remained non-significant
Firth bias-reduced logistic	Model 3	SDQ	1.144	1.067–1.232	<0.001	Full covariates including movement disability	Remained significant

B. Core indirect-effect sensitivity analyses

Analysis	Path a β (95% CI)	Path b OR (95% CI)	Total effect c OR (95% CI)	Direct effect c′ OR (95% CI)	Indirect effect (95% CI)	Adjustment set	Conclusion
Primary standard logistic	-0.830 (-1.033, -0.627)	1.155 (1.073, 1.244)	0.832 (0.741, 0.934)	0.925 (0.811, 1.056)	-0.120 (-0.193, -0.055)	Full covariates including movement disability	Indirect effect significant; direct effect non-significant
Exclude movement disability	-0.856 (-1.059, -0.654)	1.151 (1.070, 1.239)	0.831 (0.742, 0.932)	0.928 (0.814, 1.059)	-0.121 (-0.194, -0.055)	Full covariates except movement disability	Materially unchanged
Parsimonious core-confounder model	-0.911 (-1.110, -0.711)	1.132 (1.056, 1.215)	0.845 (0.759, 0.942)	0.944 (0.832, 1.071)	-0.113 (-0.186, -0.048)	Sex, age, BMI, exercise habit, only-child status	Materially unchanged
Firth bias-reduced logistic	-0.830 (-1.033, -0.627)	1.144 (1.067, 1.232)	0.841 (0.750, 0.939)	0.933 (0.820, 1.061)	-0.112 (-0.182, -0.049)	Full covariates; path a estimated by linear regression	Materially unchanged

Note. Outcome: parent- or guardian-reported school refusal tendency in 2023 (84/314 events). Primary exposure: ISI total score in 2020. Candidate intermediate variable: SDQ total difficulties in 2023. Model 1 included ISI and covariates; Model 2 included SDQ total difficulties and covariates; Model 3 included both ISI and SDQ total difficulties and covariates. Standard logistic confidence intervals are Wald intervals. Firth confidence intervals and p-values are based on profile penalized likelihood. Indirect effects are reported on the log-odds scale and were estimated using 20,000 Monte Carlo draws. The parsimonious adjustment set was defined theoretically and was not selected according to statistical significance. BMI, body mass index; CI, confidence interval; ISI, Index of Social Interaction; OR, odds ratio; SDQ, Strengths and Difficulties Questionnaire.

Table S4. Distribution, ceiling effect, and exploratory nonlinearity analyses of the ISI total score

A. Distribution summary

Statistic	Value
N	314
Mean ± SD	15.15 ± 2.28
Median (IQR)	16 (14–17)
Range	5–18
Skewness	−1.842
Score = 18	21 (6.7%)
Score = 17–18	86 (27.4%)

B. Formal nonlinearity tests with full covariate adjustment

Model/check	Nonlinear-term estimate	95% CI	Term p-value	Model-comparison p-value	Interpretation
Quadratic ordinary logistic	$\beta(ISI^2) = 0.070$	0.031–0.109	<0.001	<0.001	Evidence of curvature
Restricted cubic spline (3 df)	—	—	—	<0.001	Spline improved fit versus linear ISI term
Quadratic Firth logistic	$\beta(ISI^2) = 0.061$	0.027–0.102	<0.001	—	Curvature remained with bias-reduced estimation

C. ISI score distribution and observed school refusal tendency

ISI score	N	School refusal events	Observed event rate, %	Adjusted predicted probability
5	1	1	100.0	0.992
6	3	3	100.0	0.971
7	2	2	100.0	0.915
8	4	2	50.0	0.805
9	2	2	100.0	0.648
10	3	2	66.7	0.487
11	6	2	33.3	0.361
12	11	4	36.4	0.278
13	11	1	9.1	0.229
14	44	6	13.6	0.206
15	55	15	27.3	0.204
16	86	20	23.3	0.222
17	65	16	24.6	0.266
18	21	8	38.1	0.342

Note. The confirmed ISI total score was used. Nonlinear models used the full covariate set; the restricted cubic spline had three degrees of freedom. Model-comparison p-values were based on likelihood-ratio tests against the linear ISI model. Adjusted probabilities were averaged over the analytical sample. Because low ISI scores were sparse, these analyses should be interpreted cautiously. CI, confidence interval; df, degrees of freedom; IQR, interquartile range; ISI, Index of Social Interaction; SD, standard deviation.

Table S5. Baseline SDQ-adjusted sensitivity analyses for school refusal tendency

Analysis	Path a β (95% CI)	p	Total effect c OR (95% CI)	p	Path b OR (95% CI)	p	Direct effect c′ OR (95% CI)	p	Indirect effect (95% CI)	Interpretation
Original primary analysis (no baseline SDQ adjustment)	−0.830 (−1.033, −0.627)	<0.001	0.832 (0.741, 0.934)	0.002	1.155 (1.073, 1.244)	<0.001	0.925 (0.811, 1.056)	0.250	−0.1199 (−0.1932, −0.0555)	Significant indirect effect
Baseline SDQ-adjusted standard logistic (full covariates)	−0.554 (−0.741, −0.367)	<0.001	0.876 (0.775, 0.991)	0.035	1.129 (1.039, 1.225)	0.004	0.931 (0.816, 1.063)	0.293	−0.0670 (−0.1235, −0.0200)	Indirect effect remained significant
Baseline SDQ-adjusted Firth logistic (full covariates)	−0.554 (−0.741, −0.367)	<0.001	0.885 (0.784, 0.999)	0.048	1.118 (1.031, 1.211)	0.007	0.938 (0.823, 1.069)	0.340	−0.0617 (−0.1163, −0.0160)	Materially unchanged
Baseline SDQ-adjusted standard logistic (parsimonious covariates)	−0.605 (−0.789, −0.420)	<0.001	0.895 (0.797, 1.006)	0.064	1.107 (1.022, 1.198)	0.012	0.950 (0.837, 1.079)	0.434	−0.0614 (−0.1179, −0.0128)	Indirect effect remained significant

Note. Full covariates: sex, age, BMI, only-child status, exercise habit, good sleep, hospitalization history, parental scolding, and movement disability. Parsimonious covariates: sex, age, BMI, exercise habit, and only-child status. All baseline-adjusted models additionally included baseline SDQ total difficulties. Indirect effects were estimated on the log-odds scale using 20,000 Monte Carlo draws. Firth confidence intervals are penalized Wald intervals. BMI, body mass index; CI, confidence interval; ISI, Index of Social Interaction; OR, odds ratio; SDQ, Strengths and Difficulties Questionnaire.

Table S6. Detailed baseline SDQ-adjusted logistic regression estimates

Analysis	Model	Predictor	OR	95% CI	p-value	Adjustment set
Standard logistic	Model 1	ISI total score	0.876	0.775–0.991	0.035	Baseline SDQ + full covariates
Standard logistic	Model 1	Baseline SDQ total	1.084	1.026–1.145	0.004	Baseline SDQ + full covariates
Standard logistic	Model 2	Follow-up SDQ total	1.144	1.057–1.237	<0.001	Baseline SDQ + full covariates
Standard logistic	Model 2	Baseline SDQ total	1.043	0.979–1.112	0.196	Baseline SDQ + full covariates
Standard logistic	Model 3	ISI total score	0.931	0.816–1.063	0.293	Baseline SDQ + full covariates
Standard logistic	Model 3	Follow-up SDQ total	1.129	1.039–1.225	0.004	Baseline SDQ + full covariates
Standard logistic	Model 3	Baseline SDQ total	1.040	0.976–1.108	0.229	Baseline SDQ + full covariates
Firth bias-reduced	Model 1	ISI total score	0.885	0.784–0.999	0.048	Baseline SDQ + full covariates
Firth bias-reduced	Model 2	Follow-up SDQ total	1.132	1.049–1.223	0.002	Baseline SDQ + full covariates
Firth bias-reduced	Model 3	ISI total score	0.938	0.823–1.069	0.340	Baseline SDQ + full covariates
Firth bias-reduced	Model 3	Follow-up SDQ total	1.118	1.031–1.211	0.007	Baseline SDQ + full covariates
Firth bias-reduced	Model 3	Baseline SDQ total	1.039	0.976–1.106	0.233	Baseline SDQ + full covariates
Standard logistic	Parsimonious Model 1	ISI total score	0.895	0.797–1.006	0.064	Baseline SDQ + core covariates
Standard logistic	Parsimonious Model 3	ISI total score	0.950	0.837–1.079	0.434	Baseline SDQ + core covariates
Standard logistic	Parsimonious Model 3	Follow-up SDQ total	1.107	1.022–1.198	0.012	Baseline SDQ + core covariates
Standard logistic	Parsimonious Model 3	Baseline SDQ total	1.038	0.974–1.106	0.256	Baseline SDQ + core covariates

Note. Full covariates included sex, age, BMI, only-child status, exercise habit, good sleep, hospitalization history, parental scolding, and movement disability. Core covariates included sex, age, BMI, exercise habit, and only-child status. Standard logistic confidence intervals are Wald intervals; Firth confidence intervals are penalized Wald intervals.

Table S7. Domain-specific indirect-association analyses adjusted for the corresponding baseline SDQ domain

Candidate domain	Path a β (95% CI)	p	Path b OR (95% CI)	p	Total effect c OR (95% CI)	p	Direct effect c′ OR (95% CI)	p	Indirect effect (95% CI)	Significant indirect effect
Emotional symptoms	−0.158 (−0.229, −0.088)	<0.001	1.395 (1.139, 1.708)	0.001	0.848 (0.752, 0.957)	0.007	0.885 (0.779, 1.005)	0.060	−0.0526 (−0.0973, −0.0176)	Yes
Conduct problems	−0.132 (−0.193, −0.071)	<0.001	1.060 (0.840, 1.337)	0.622	0.839 (0.744, 0.945)	0.004	0.846 (0.747, 0.957)	0.008	−0.0077 (−0.0417, 0.0236)	No
Hyperactivity/inattention	−0.180 (−0.259, −0.102)	<0.001	1.011 (0.845, 1.211)	0.903	0.840 (0.747, 0.946)	0.004	0.842 (0.745, 0.952)	0.006	−0.0020 (−0.0367, 0.0318)	No
Peer problems	−0.095 (−0.165, −0.026)	0.007	1.430 (1.156, 1.768)	<0.001	0.851 (0.754, 0.961)	0.009	0.871 (0.769, 0.987)	0.030	−0.0341 (−0.0712, −0.0068)	Yes
Prosocial behavior	0.053 (−0.028, 0.133)	0.201	0.814 (0.675, 0.980)	0.030	0.843 (0.749, 0.948)	0.004	0.852 (0.755, 0.961)	0.009	−0.0108 (−0.0357, 0.0055)	No
Internalizing problems	−0.254 (−0.369, −0.139)	<0.001	1.315 (1.151, 1.501)	<0.001	0.862 (0.761, 0.976)	0.019	0.904 (0.791, 1.033)	0.139	−0.0694 (−0.1210, −0.0288)	Yes
Externalizing problems	−0.297 (−0.415, −0.180)	<0.001	1.020 (0.904, 1.152)	0.746	0.845 (0.749, 0.953)	0.006	0.850 (0.749, 0.964)	0.011	−0.0060 (−0.0446, 0.0309)	No

Note. Each model adjusted the follow-up SDQ domain for the corresponding baseline domain and the full covariate set. Indirect effects were estimated on the log-odds scale using 20,000 Monte Carlo draws. These analyses were exploratory and were not adjusted for multiple comparisons. CI, confidence interval; OR, odds ratio; SDQ, Strengths and Difficulties Questionnaire.

Table S8. Baseline SDQ-adjusted Firth analyses for frequent school absence

Model	Predictor	OR	95% CI	p-value	Indirect effect (95% CI)	Interpretation
Model 1	ISI total score	0.694	0.567–0.850	<0.001	—	Total association significant
Model 2	Follow-up SDQ total	1.424	1.169–1.735	<0.001	—	Association significant
Model 3	ISI total score	0.816	0.647–1.029	0.086	−0.1350 (−0.2675, −0.0229)	Direct association non-significant
Model 3	Follow-up SDQ total	1.276	1.045–1.558	0.017	−0.1350 (−0.2675, −0.0229)	Indirect effect significant
Model 3	Baseline SDQ total	1.083	0.933–1.258	0.295	—	Baseline SDQ non-significant

Note. Frequent school absence occurred in only 16 participants. Models used Firth bias-reduced logistic regression and adjusted for baseline SDQ total difficulties plus the full covariate set. Confidence intervals are penalized Wald intervals. The indirect effect was estimated with 20,000 Monte Carlo draws and should be regarded as supplementary exploratory evidence. CI, confidence interval; ISI, Index of Social Interaction; OR, odds ratio; SDQ, Strengths and Difficulties Questionnaire.

Table S9. Data and model diagnostics for baseline SDQ-adjusted analyses

A. Data and model diagnostics

Diagnostic	Value	Reference/Interpretation	Conclusion
Analytical sample	314	All baseline and follow-up variables complete	No sample-size reduction
School refusal tendency events	84	Primary binary outcome	26.8%
Frequent school absence events	16	Supplementary rare outcome	Firth regression used
Baseline SDQ missing	0	Baseline total and all domains	Complete
Baseline SDQ total, mean ± SD	10.15 ± 5.34	Range 1–27	—
Follow-up SDQ total, mean ± SD	9.07 ± 4.61	Range 0–28	—
Baseline–follow-up SDQ correlation	0.568	Pearson correlation	Moderate correlation
Maximum VIF in baseline-adjusted Model 3	1.82	<5 commonly considered acceptable	No meaningful multicollinearity
Events per predictor parameter	7.0	12 predictor parameters in full Model 3	Sensitivity and Firth analyses warranted
SDQ change-score association with ISI	−0.110 (−0.347, 0.128)	p = 0.365	Not statistically significant

B. Variance inflation factors in the full baseline SDQ-adjusted Model 3

Predictor	VIF	Interpretation
ISI total score	1.349	<5
Follow-up SDQ total difficulties	1.817	<5
Baseline SDQ total difficulties	1.544	<5
Sex	1.071	<5
Age	1.166	<5
BMI	1.093	<5
Only-child status	1.042	<5
Exercise habit	1.080	<5
Good sleep	1.116	<5
Hospitalization history	1.049	<5
Parental scolding	1.031	<5
Movement disability	1.059	<5

Note. The change-score analysis regressed follow-up minus baseline SDQ total difficulties on baseline ISI and the full covariate set. BMI, body mass index; ISI, Index of Social Interaction; SD, standard deviation; SDQ, Strengths and Difficulties Questionnaire; VIF, variance inflation factor.
